# Proximal gastrectomy and double-tract reconstruction vs total gastrectomy in gastric and gastro-esophageal junction cancer patients — a systematic review and meta-analysis protocol (PROSPERO registration number: CRD42021291500)

**DOI:** 10.1186/s13643-023-02304-3

**Published:** 2023-08-29

**Authors:** Julian Hipp, Jasmina Kuvendjiska, Verena Martini, Hans Christian Hillebrecht, Stefan Fichtner-Feigl, Markus K. Diener

**Affiliations:** Department of General and Visceral Surgery, Medical Center-University of Freiburg, Faculty of Medicine-University of Freiburg, Hugstetter Str. 55, 79106 Freiburg, Germany

**Keywords:** Systematic review, Meta-analysis, Protocol, Gastric cancer, Esophagogastric junction cancer, Proximal gastrectomy, Double-tract reconstruction, Gastrectomy, Visceral surgery, Oncologic surgery

## Abstract

**Background:**

In Germany and Western Europe, gastroesophageal junction cancer (AEG) and proximal gastric cancer are currently treated with (transhiatal-extended) total gastrectomy (TG) according to the latest treatment guidelines. TG leads to a severe and long-lasting impairment of postoperative health-related quality of life (HRQoL) of the treated patients. Recent studies have suggested that HRQoL of these patients could be improved by proximal gastrectomy with double-tract reconstruction (PG-DTR) without compromising oncologic safety. Our aim is therefore to conduct a randomized controlled non-inferiority trial comparing PG-DTR with TG in AEG II/III and gastric cancer patients with overall survival as primary endpoint and HRQoL as key secondary endpoint.

**Methods:**

This protocol is written with reference to the Preferred Reporting Items for Systematic Review and Meta-Analysis Protocols (PRISMA-P 2015) statement. We will conduct searches in the electronic databases MEDLINE, Web of Science Core Collection, ScienceDirect, and Cochrane Library. We will also check references of relevant studies and perform a cited reference research. Titles and abstracts of the records identified by the searches will be screened, and full texts of all potentially relevant articles will be obtained. We will consider randomized trials and non-randomized studies. The selection of studies, data extraction, and assessment of risk of bias of the included studies will be conducted independently by two reviewers. Meta-analysis will be performed using RevMan 5.4 (Review Manager (RevMan) Version 5.4, The Cochrane Collaboration).

**Discussion:**

This systematic review will identify the current study pool concerning the comparison of TG and PG-DTR and help to finally refine the research questions and to allow an evidence-based trial design of the planned multicenter randomized-controlled trial.

**Ethics and dissemination:**

Ethical approval is not required for this systematic review. Study findings will be shared by publication in a peer-reviewed journal.

**Systematic review registration:**

PROSPERO CRD42021291500.

**Supplementary Information:**

The online version contains supplementary material available at 10.1186/s13643-023-02304-3.

## Background

Upper gastrointestinal tract cancers originating in the gastro-esophageal junction (AEG) or the stomach are one of the most frequent reasons for cancer-related death worldwide. In Germany, AEG and gastric cancer are the 5th (men) and 6th (women) most commonly diagnosed cancer. AEG cancers are showing a dramatic increase in incidence, while gastric cancer of the distal part of the stomach is becoming less common in western countries [1]. For early gastric cancer without risk factors (T1a/b sm1), endoscopic resection with endoscopic submucosal dissection (ESD) or endoscopic mucosa resection (EMR) is an option. Standard of surgical treatment for all other patients with resectable gastric cancer in the upper third of the stomach and gastro-esophageal junction (AEG)-cancer type Siewert II and III [2] is total gastrectomy (TG) or transhiatal-extended gastrectomy. The surgical treatment is combined with perioperative chemotherapy in locally advanced cases [3, 4].

Total gastrectomy significantly impairs long-term health-related quality of life (HRQoL) of the patients. Compared to subtotal-distal gastrectomy, HRQoL of patients with TG is substantially impaired for physical and role functioning, appetite loss, and eating restrictions [5]. The nutritional status and the HRQoL of patients undergoing subtotal gastrectomy have been shown to be superior compared to TG, while both procedures offer equal overall survival rates in one randomized controlled trial (RCT), provided that the proximal margin of resection falls in healthy tissue [6]. As the results of subtotal-distal gastrectomy are superior to TG, efforts have been made to develop an organ-preserving approach for proximal gastric cancer and AEG cancers as well. Proximal gastrectomy offers similar survival rates compared to TG in retrospective studies, while HRQoL appeared to be improved [7]. Proximal gastrectomy procedure includes D1 and D2 lymphadenectomy and resection of the upper two-thirds of the stomach and the distal esophagus, previously followed by esophagogastrostomy with gastric tube reconstruction or Merendino reconstruction with jejunal interposition [8]. However, this leads to a severely increased rate of gastroesophageal reflux with reflux esophagitis and anastomotic stricture [9]. A solution to the functional problems has been found with double-tract reconstruction after proximal gastrectomy (PG-DTR) [10–12]. PG-DTR is a proximal gastrectomy with preservation of the distal stomach. Double-tract reconstruction is performed after standardized Roux-en-Y reconstruction with an additional side-to-side anastomosis of the distal stomach to the alimentary limb of the Roux-en-Y reconstruction. According to the literature, this procedure has a lower rate of postoperative reflux esophagitis and other beneficial long-term effects compared to TG including a reduced postoperative loss of body weight and improved hemoglobin, albumin, and vitamin B12 levels [13, 14] with a similar rate of postoperative complications and long-term overall survival [15, 16].

### Objectives

As there seems to be a complete lack of RCTs comparing PG-DTR and TG in a prospective, randomized trial with oncologic endpoint (overall survival, disease-free/local recurrence-free survival) and HRQoL as endpoint, we aim to perform this RCT to assess the comparative effectiveness of PG-DTR according to the IDEAL framework for surgical innovation [17]. Within the project development phase for this trial, a systematic review and meta-analysis is necessary to finally define and adapt the research question including the design and methodology of the planned RCT taking into account the findings.

The review will address the following questions:

1. Is PG-DTR non-inferior compared to TG with regard to overall survival according to the current study pool?
2. Is PG-DTR non-inferior compared to TG with regard to disease-free survival/local recurrence-free survival according to the current study pool?
3. Is PG-DTR superior to TG with regard to HRQoL?
4. In what populations/settings has PG-DTR been evaluated?
5. What patients are feasible for randomization between PG-DTR and TG?
  - With regard to TNM stage/neoadjuvant treatment.
  - With regard to tumor localization in the stomach (minimal distance to the pylorus).
6. Which is the necessary size/volume of the remnant stomach for improvement of HRQoL postoperatively compared to TG/is TG with double-tract reconstruction feasible and beneficial?
7. Which are the chosen distances between esophagojejunostomy and jejunogastrostomy and Roux-en-Y jejunojejunostomy in the current study pool? Is HRQoL impaired by the distances between the anastomoses?
8. Which outcomes have been addressed in the current study pool?

## Methods and analysis

This protocol is written with reference to the Preferred Reporting Items for Systematic Review and Meta-Analysis Protocols (PRISMA-P 2015) statement [18] (Supplementary Data).

### Eligibility criteria

#### Participants/population

We will focus on studies including patients with resectable non-metastatic gastric cancer and gastro-esophageal junction cancer (AEG-carcinoma) type II and III [2]. Studies including patients with and without multimodal treatment/(neo-)adjuvant treatment will be included.

### Intervention and comparator treatment

We will consider proximal gastrectomy with double-tract reconstruction (PG-DTR) as eligible intervention. Total gastrectomy (TG) will be the comparator treatment. We will consider open-surgical and laparoscopic/robotic approaches as eligible treatments.

### Outcomes

The following outcomes will be extracted.

- Overall survival
- Disease-free survival
- Local recurrence-free survival
- Quality of life
- Postoperative weight loss/changes in body-mass index
- Frequency of postoperative anemia
- Postoperative changes in the following: serum-hemoglobin, serum-iron, serum-vitamin b12, serum-albumin, serum-total-protein, and serum-total-cholesterol.

This list of outcomes is non-exhaustive and will be completed depending on the outcomes reported in the identified study pool.

### Study types

Prospective randomized controlled trials (RCTs), prospective studies without randomized patient allocation (non-randomized controlled studies/NRS), and retrospective observational studies (with control group) will be eligible for the systematic review and meta-analysis. We will not consider single-arm studies due to the missing control group in this study design. The reason for this exclusion is that studies without a control group provide no reliable data to estimate comparative effectiveness and will not be useful for the meta-analysis and the planned randomized controlled trial. Furthermore, review articles, clinical guidelines, and work that have not been peer reviewed will be excluded. We will not apply any exclusion criteria regarding study duration and setting. We will only consider studies written in English or German language.

### Information sources

The searches for this systematic review will be performed and conducted by following the recommendations of PRESS (Peer Review of Electronic Search Strategies) [19]. We will not use any date restrictions in the electronic searches. For each database, the date of search, the search strategy, and the number of results will be documented. Systematic searches will be conducted in the following electronic data sources:

- MEDLINE, MEDLINE Daily Update, MEDLINE In-Process and Other Non-Indexed Citations, MEDLINE Epub Ahead of Print (via Ovid) (a preliminary search strategy is displayed in Table 1).
- Web of Science Core Collection: Science Citation Index-EXPANDED (SCI-EXPANDED) (via Clarivate Analytics).
- Cochrane Library (via Wiley).
- ScienceDirect (via Elsevier).
- Searches for unpublished and ongoing studies will be performed in ClinicalTrials.gov (www.clinicaltrials.gov) and WHO International Clinical Trials Registry Platform (http://www.who.int/ictrp/search/en) and the German study register (www.drks.de).

**Table 1:** Preliminary search strategy for MEDLINE

Search	Query	Results
#7	Search: #6 NOT (animals [mh] NOT humans [mh])	924
#6	Search: (#1 AND ((#2 AND #3) OR #4)) AND #5	929
#5	Search: random*[tiab] OR RCT[tiab] OR “Randomized Controlled Trial”[pt] OR “Randomized Controlled Trials as Topic”[Mesh] OR “Clinical Trial”[pt] OR “Clinical Study” [pt] OR “Controlled Clinical Trials as Topic”[Mesh] OR “Observational study” [pt] OR “Comparative Study” [Publication Type] OR “Multicenter Study”[Publication Type] OR “controlled study”[tiab] OR group*[tiab] OR cohort*[tiab] OR “Control Groups”[Mesh] OR “Prospective Studies”[Mesh] OR control[tiab] OR controls[tiab] OR versus[tiab] OR compar*[tiab] OR matched[tiab]	11,653,482
#4	Search: “double tract”[tiab] or “double-tract”[tiab] or “two tract”[tiab] or “two-tract”[tiab] OR DTR [tiab]	1113
#3	Search: proximal[tiab]	224,119
#2	Search: gastrectom*[tiab] OR “Gastrectomy”[Mesh]	49,888
#1	Search: ((gastric*[tiab] OR stomach[tiab] OR gastrointestinal*[tiab] OR gastro-oesophageal*[tiab] OR gastrooesophageal*[tiab] OR gastro-esophageal*[tiab] OR gastroesophageal*[tiab] OR esophagogastric*[tiab]) AND (cancer[tiab] OR carcinoma*[tiab] OR adenocarcinoma*[tiab] OR neoplas*[tiab] OR tumor[tiab] OR tumors[tiab] OR tumour*[tiab] OR malignan*[tiab])) OR “Stomach Neoplasms”[Mesh]	218,033

We will use relevant studies and/or systematic reviews to search for additional references via the PubMed similar articles function and forward citation tracking. Reference lists of relevant articles will also be reviewed manually.

### Identification of relevant studies

Titles and abstracts of records identified by the searches will be screened, and full text of all potentially relevant articles will be obtained. Full texts will be checked for eligibility by two independent reviewers, and reasons for exclusion will be documented (full-text screening).

### Extraction of study data

For quality assurance purposes, two independent reviewers will extract the following study data independently in duplicate into a predefined data-extraction table. A third reviewer will resolve discrepancies between the two reviewers.

- Study characteristics: Title, author, year of publication, journal, language, setting (geographical), trial duration, trial design (RCT/NRS/observational study), eligible diseases (esophageal cancer/gastric cancer), eligible tumor stages of patients according to the TNM-classification, total number of patients, number of treatment groups, and patients per group (matched cohorts).
- Patient characteristics: Age, gender, disease (esophageal cancer/gastric cancer), tumor stage (TNM stage of patients included to the study), multimodal treatment/(neo-)adjuvant treatment, operative time (minutes), number of harvested lymph nodes, length of postoperative hospital stay (days), complications according to Clavien-Dindo classification [20], incidence of anastomotic leakages, incidence of reflux esophagitis according to the Los Angeles classification [21], incidence of anastomotic stricture, and duration of follow-up (months).
- Outcome parameters: As previously mentioned.

### Risk-of-bias assessment

The risk of bias (RoB) will be assessed using the Revised Cochrane risk-of-bias tool for randomized trials (RoB 2) [22] and the ROBINS-I tool developed by the Cochrane Bias Methods Group [23], as applicable, by two independent reviewers. The Revised Cochrane risk-of-bias tool (RoB 2) includes five standard domains of bias: bias arising from the randomization process, bias due to deviations from intended interventions, bias due to missing outcome data, bias in measurement of the outcome, and bias in selection of the reported result. Each domain will be judged to be “low risk of bias,” “some concerns,” or “high risk of bias.” The ROBINS-I-tool covers seven domains through which bias might be introduced into a non-randomized study: bias due to confounding, bias in selection of participants into the study, bias in classification of interventions, bias due to deviations from intended interventions, bias due to missing data, bias in measurement of outcomes, and bias in selection of the reported result. The response options for each domain level are as follows: “low risk of bias,” “moderate risk of bias,” “serious risk of bias,” “critical risk of bias,” and “no information.”

### Statistical analysis

Regarding the main research questions of the planned systematic review, the following questions will be handled in form of a narrative review: in what populations/settings has PG-DTR been evaluated? What patients are feasible for randomization between PG-DTR and TG? Which is the necessary size/volume of the remnant stomach for improvement of HRQoL postoperatively compared to TG/is TG with double-tract reconstruction feasible and beneficial? Which are the chosen distances between esophagojejunostomy and jejunogastrostomy and Roux-en-Y jejunojejunostomy in the current study pool? Is HRQoL impaired by the distances between the anastomoses? Depending on the available data, quantitative analysis may be performed regarding differences in HRQoL data. Otherwise, a narrative approach will be applied. Survival data and nutritional parameters will be assessed with a quantitative approach.

Statistical analysis will be performed with the Review Manager (RevMan) Version 5.4.1 (The Cochrane Collaboration, The Nordic Cochrane Centre, Copenhagen, Denmark) [24]. Relevant outcome parameters from the included trials will be assessed for estimation of treatment effects, if data can be synthesized. In the case that quantitative analysis is not possible, we will summarize the collected study data by means of a narrative review using tables and figures (e.g., bubble plots) to present and explain the research landscape and to describe potential research clusters and/or gaps in this patient population.

For meta-analysis, odds ratios and associated 95% confidence intervals will be calculated for dichotomous data by Mantel–Haenszel or inverse-variance models, as applicable. Weighted mean differences (MDs) and associated 95% confidence intervals will be calculated using inverse-variance models. Time-to-event data will be estimated when necessary with indirect methods [25] and analyzed by “O–E and variance” outcome type, and results will be expressed as Peto odds ratio. When necessary, missing standard deviations will be obtained from standard errors, confidence intervals, *t*-values, and *p*-values [26]. For all statistical analyses, a two-sided *p*-value < 0.05 will be considered statistically significant.

We will assess the heterogeneity of effects across studies using the *I*^2^ statistics. An *I*^2^ value of > 50% will be considered an indication of substantial heterogeneity [24]. We will use the random-effects model as the interventions and populations are likely to be heterogeneous across included studies as the default model for meta-analysis, if there is no indication of funnel plot asymmetry. In case of funnel-plot asymmetry, we will present both analyses or neither (in which case a narrative review of the data will be presented), according to Cochrane Handbook for Systematic Reviews of Interventions. To investigate the risk of population bias, funnel plots will be generated for meta-analyses and tested for asymmetry with the Harbord test [27]. Subgroup analyses of patients with early gastric cancer (T1N0) and patients with locally advanced gastric cancer (≥ T2 and/or N +) are planned. Rating of quality of evidence will be performed for each outcome variable using the GRADE approach. Although non-randomized studies of interventions will be included, all findings will start as high certainty of evidence because their RoB will be assessed using the ROBINS-I tool. After the initial level of certainty is established, we will judge the quality of evidence based on the suggested five criteria for down-rating our confidence in effects estimates (risk of bias, inconsistency, imprecision, indirectness, and publication bias) and the three criteria for uprating our confidence (large effect, dose–response gradient and opposing confounding) [28]. The different bodies of evidence will be dealt with according to Cuello-Garcia et al. [29]. If necessary, two distinct tables for the rating of quality of evidence will be integrated to the systematic review.

## Discussion/perspective

Currently in Germany and Western Europe, patients with AEG II and III tumors and proximal gastric cancer are treated with (transhiatal-extended) TG. TG leads to a severe impairment of postoperative HRQoL. Recent studies have suggested that HRQoL of these patients could be improved by PG-DTR without compromising oncologic safety. This leads to an ethical need to further investigate this surgical procedure and demonstrate its oncological safety and improved HRQoL by means of a randomized controlled trial comparing PG-DTR with the standard treatment TG.

To support the planned clinical trial, this systematic review will identify the current study pool concerning the comparison of TG and PG-DTR and help to finally refine the research questions and to allow a good and evidence-based trial design. With the results of this systematic review and meta-analysis, adequate sample size calculation and choice of important endpoints for the planned RCT will be possible. We believe that with the results of our RCT, substantial progress can be achieved by optimization of the surgical treatment with prospective and randomized validation for AEG and gastric cancer patients.

### Supplementary Information

**Additional file 1.** 
